# Housing adaptations and older adults’ health trajectories by level of initial health: evidence from the English Longitudinal Study of Ageing

**DOI:** 10.1093/ageing/afaf023

**Published:** 2025-02-17

**Authors:** Jiawei Wu, Emily M Grundy

**Affiliations:** Asian Demographic Research Institute, Shanghai University, Shanghai, China; ISER, University of Essex, Colchester CO4 3SQ, UK

**Keywords:** housing adaptations, ADL (activities of daily living), IADL (instrumental activities of daily living), ELSA (English Longitudinal Study of Ageing), older people

## Abstract

**Background:**

In many ageing societies, the housing stock is poorly designed to meet the needs of older people with health limitations. Housing adaptations may enable older people to retain functional ability in the home, improve well-being and reduce the risks of falls. There is mixed evidence on whether adaptations are most beneficial for those who have limitations or whether they have a greater impact if implemented before people experience substantial disability. This study aimed to identify socio-demographic factors associated with obtaining housing adaptations and whether and how the impact of adaptations on changes in mental and physical health varied by initial level of health measured using objective indicators.

**Methods:**

We used data from the English Longitudinal Study of Ageing to analyse socio-demographic factors associated with acquiring housing adaptations using logistic regression. We then estimated mixed-effects models to assess how a measure of baseline physical health, derived from observer-measured indicators of physical function, modified the association between acquiring housing adaptations and health and disability outcomes for two cohorts each followed up for three waves.

**Results:**

Having more activities of daily living (ADL) limitations was positively associated with acquiring housing adaptations, but we found no evidence for socio-demographic variations. Acquiring housing adaptations was associated with slower development of instrumental ADL/ADL disability among older people with initially good latent physical health. Sensitivity analysis suggested that housing adaptations mitigated the predicted probability of falls for those with severe mobility impairments.

**Conclusions:**

Housing adaptations may slow down development of disability in older people with initially good health.

## Key Points

- Having more activities of daily living (ADL) limitations was positively associated with acquiring housing adaptations.
- No consistent socio-economic gradient by education, tenure or wealth in acquiring housing adaptations.
- Acquiring housing adaptations was associated with slower instrumental ADL/ADL deterioration for those with initially good latent health.

## Introduction

The home environment is an important influence on older people’s health and quality of life [1, 2] and may affect their ability to undertake everyday activities and fulfil social roles [3]. However, in many ageing societies, housing conditions are ill-suited to meet the needs of older people with health limitations. The UK has a higher proportion of pre-1946 housing stock than any European Union country [4], with only 9% of homes in England meeting prescribed accessibility standards in 2018 [5] and 15% of older households in 2020–21 lived in homes that failed to meet the minimum standards on absence of hazards, thermal comfort, state of repair and facilities specified for new dwellings [6]. Although increases in new build specialist housing for older people have been advocated, currently only 10% of older households live in such housing [7] and most older people want to remain in their long-term homes [8]. Housing adaptations have been suggested as means of improving older adults’ living conditions, safety and functional ability at home [9]. However, evidence on the effectiveness of these as a means of mitigating long-term conditions is mixed and there is conflicting evidence on whether its effectiveness as a preventive or enabling strategy varies by initial health and disability status.

In England, local authorities are responsible for providing minor adaptations (such as the installation of grab rails) free of charge for older people who need them after an assessment by an occupational therapist. Means-tested funding for more substantial adaptations is available through Disabled Facilities Grants (DFGs). However, there are substantial variations between local authorities in the help provided and often long delays in accessing services, which may mean that older people with sufficient resources decide to pay for modifications privately [2, 8, 10, 11]. Information on socio-demographic variations in obtaining housing adaptations is, however, sparse.

Some UK studies of specific groups have shown that the installation of housing adaptations enabled older adults to stay in their current home longer [12] and reduced care home admissions for moderately and severely frail older adults, although it increased them for the less frail [13]. Many randomised controlled trials (RCTs) [14] have found that housing adaptations protected against falls [15–17], fall-related injuries [18], and mortality [19]. However, RCTs often suffer from short follow-up periods and sometimes nonrepresentative samples and evidence from systematic reviews is inconsistent [20]. Analyses of representative panel data suggest that housing adaptations mitigated the decline in older Americans’ physical function [21] and buffered the negative effects of mobility impairments on falls, pain and poor health among older adults in England [22]. One recent systematic review of RCTs only found a reduction in falls among people selected for a higher risk of falling [23]. Other studies have suggested that household environmental hazards may pose the greatest risk for older people with fair rather than poor or good health [24]. It is thus unclear whether adaptations are most useful for those with existing disabilities or whether they also have preventive effects in slowing down the development of disability in those with no or few functional limitations.

In this study, we address two questions using nationally representative panel data for England. Firstly, we investigate health-related and socio-demographic factors associated with acquiring housing adaptations. Secondly, we examine how baseline physical health (a categorical latent variable derived from objective indicators measured by nurses) moderates the association between acquiring housing adaptations and changes in older adults’ health and well-being. We use gender-stratified samples due to the well-documented female disadvantage in physical health [25].

## Materials and methods

We used data from Waves 2 to 9 of the English Longitudinal Study of Ageing (ELSA) [26]. The first wave of ELSA (2002/03) had a response rate of 67%, including 11 391 core members who have been followed up every 2 years. The sample has been regularly refreshed, including at Wave 6. ELSA collects information on demographic, socio-economic and health characteristics. Nurse visit data, which we used to assess older adults’ latent physical health, were first collected at Wave 2 and subsequently at Waves 4, 6, 8 and 9. The questions about housing adaptations were the same across Waves 2–5 but changed substantially from Wave 6 onwards. Therefore, we distinguished earlier (w2–5) and later (w6–9) cohorts. We focused on ELSA core members aged 65+ at respective baseline waves (i.e. Wave 2 or 6) who at that point had no housing adaptations and who were re-interviewed at least once across Waves 3–5 or 7–9. The final analytic sample comprised 2587 participants (1224 men and 1363 women) for Waves 2–5 and 2123 participants (983 men and 1140 women) for Waves 6–9. We conducted a complete case analysis, so the observations included in regression models vary slightly due to differences in response to particular questions.

### Measures

For our first research question, the outcome is whether participants reported acquiring housing adaptations during the follow-up period. At Waves 2–5, respondents were asked if they had the following home adaptations: widened doorways/hallways; ramps or street-level entrances; handrails; automatic or easy open doors; accessible parking; bathroom modifications; kitchen modifications; lift, chair lift or stair glide; alerting devices; and any other special features. In Waves 6–9, the items further included bed lever/rail, hoist and toilet equipment/commode while bathroom modifications were broken down to walk-in shower, over-bath shower and bath/shower seat. We excluded the item about the over-bath shower as this is a common feature in contemporary homes and not necessarily an adaptation prompted by disability and derived a binary outcome indicating whether respondents had acquired any housing adaptations by last observation point. Participants’ age, latent physical health (bad, fair, good), number of limitations of activities of daily living (ADL) (dressing, walking across the room, bathing, eating, getting in or out of bed, and using the toilet), number of limitations of instrumental activities of daily living (IADL) (preparing meals, shopping for groceries, making phone calls, taking medications, doing housework and managing money), housing tenure, partnership status, educational attainment and wealth (defined as the Benefit Unit’s total nonpension wealth) were predictors considered. The categorical physical health measure was constructed based on a continuous latent physical health score derived from grip strength, full tandem stand, inversed chair rise time and lung function in a structural equation modelling framework [27] for men and women separately at Waves 2 and 6 (Supplementary Materials Table S1 and Fig. S1).

For the second research question, informed by previous studies [21, 22], our outcomes were four continuous measures: the Control, Autonomy, Self-realisation and Pleasure (CASP)-19 scale, a validated measure of quality of life ranging from 0 to 57 with higher values indicating better well-being [28, 29]; a depression score derived from the 8-item Centre for Epidemiologic Studies Depression (CESD-8) scale [30]; and a number of IADL and ADL limitations. We included IADL limitations because adaptations may render IADL tasks such as housework less demanding and be particularly helpful for people with sensory impairments [31]. We also examined a binary outcome: falls (if fell in the last 2 years, no = 0). We used the categorical latent physical health noted previously as the moderator in the main analyses. Covariates included age, age squared, marital status, education, housing tenure and wealth quintile.

### Analytical strategy

We fitted logistic regression models to analyse factors associated with acquiring housing adaptations. Depending on the nature of the outcome, we estimated mixed-effects linear or logistic models [32] to examine whether baseline latent physical health modified associations between acquiring housing adaptations and outcomes [in models without interaction terms acquiring housing adaptations was associated with worse health outcomes, while better latent health was associated with better outcomes (results not shown)]. There were three levels in the models, i.e. repeat observations across waves clustered within participants who were clustered within latent physical health tertile (poor, fair, good) at the respective baseline wave. A random slope for housing adaptations was included only in the models with continuous outcomes due to nonconvergence possibly caused by very small random slope variances when the outcome is binary [33]. Longitudinal weights were not applied because not all participants were interviewed consecutively. We conducted analyses for men and women from earlier and later cohorts separately using Stata 16.

## Results

Table 1 presents descriptive statistics for the four subsamples. The average age at baseline waves was around 72, and some 90% were owner-occupiers. This is higher than the proportion of owner-occupiers among those aged 65 and over recorded in the 2001 and 2011 Censuses for England (72% and 78%, respectively) [34, 35]. However, Table 1 excludes those who already had housing adaptations. When we dropped this criterion, the proportion of owner-occupiers in our sample was 81% [95% confidence interval (CI): 80%–82%] at Wave 2 (2004–05) and 86% (95% CI: 85% to 87%) at Wave 6 (2012–13), closer to, although still higher than, results from the Census. Educational attainment was higher among men and in the later cohort. Three-quarters of men and just over half of women were partnered. Compared with men, women reported poorer physical functioning, more depressive symptoms and a higher proportion who had had one or more falls in the previous 2 years. There were no marked gender differences in the CASP score.

**Table 1:** Socio-demographic and health characteristics of sample members at study baseline points (Waves 2 or 6); ELSA respondents aged 65 and over with no housing adaptions at baseline point and with at least one follow-up in the relevant observation window (Waves 2–5 or 6–9).

	Wave 2				Wave 6			
	Men		Women		Men		Women	
	Mean/%	SD/*n*	Mean/%	SD/*n*	Mean/%	SD/*n*	Mean/%	SD/*n*
CASP (range 0–57)	43.78	8.15	43.85	8.16	41.46	8.12	41.12	8.72
CES-D score (range 0–8)	1.06	1.57	1.65	1.95	0.83	1.39	1.32	1.78
ADL limitations (range 0–6)	0.24	0.65	0.28	0.72	0.16	0.58	0.21	0.65
IADL limitations (range 0–6)	0.18	0.59	0.27	0.71	0.17	0.58	0.22	0.70
Mobility impairments (range 0–10)	1.37	1.91	2.10	2.25	1.14	1.78	1.85	2.28
Fall in the last 2 years (no = 0)	0.24	0.43	0.34	0.47	0.24	0.43	0.27	0.44
Age	72.73	6.02	73.09	6.30	72.42	5.98	72.67	6.07
Marital status								
Never married	4.33	53	3.89	53	4.88	48	3.07	35
Married	75.00	918	51.25	698	74.36	731	55.75	635
Divorced/separated	6.37	78	8.22	112	9.56	94	13.35	152
Widowed	14.30	175	36.64	499	11.19	110	27.83	317
Education								
Lower/none	35.79	437	48.86	665	22.86	224	34.81	394
Secondary	30.30	370	19.99	272	31.22	306	28.80	326
Higher	27.93	341	17.19	234	38.37	376	21.20	240
Foreign/other	5.98	73	13.96	190	7.55	74	15.19	172
Tenure								
Owner-occupier	89.55	1080	86.78	1162	90.18	872	88.40	983
Social renter	8.37	101	11.05	148	7.55	73	8.63	96
Private renter	2.07	25	2.17	29	2.28	22	2.97	33
*N*	1224		1363		983		1140	

Note: Data source: ELSA w2–9. CASP, Control, Autonomy, Self-realisation and Pleasure; CES-D, Centre for Epidemiological Studies-Depression scale; ADL, Activities of Daily Living; IADL, Instrumental Activities of Daily Living; SD, standard deviation.

Table 2 shows the proportions of respondents who had acquired housing adaptations by the last observation point. The proportions were slightly higher for women than men and considerably higher in the later cohort.

**Table 2:** Proportion (%) with housing adaptations at the last observation point (Waves 3–5 or 7–9) among those with no housing adaptations at first observation (Wave 2 or 6), by gender.

	Men		Women	
Last observation point	% with adaptation	*N*	% with adaptation	*N*
Earlier cohort				
Wave 3	23.72	156	22.37	152
Wave 4	30.43	161	33.75	160
Wave 5	23.26	907	29.40	1051
Total	24.26	1224	29.13	1363
Later cohort				
Wave 7	31.96	97	44.62	130
Wave 8	56.64	143	46.97	132
Wave 9	56.93	743	59.91	878
Total	54.43	983	56.67	1140

Note: Data source: English Longitudinal Study of Ageing Waves 2–9.

Table 3 presents results from logistic regression models of associations between baseline characteristics and acquiring housing adaptations. Older age was positively associated with acquiring housing adaptations for men and women in both cohorts. We found no evidence of consistent variation by wealth quintile or educational level. Being a renter was associated with substantially higher odds (OR = 2.996, 95% CI: 1.428–6.285) of acquiring housing adaptations for women in the earlier cohort, but the effects were not significant for women in the later cohort or for men. More ADL limitations, but not poor latent physical health, were generally positively associated with acquiring adaptations.

**Table 3:** Results from logistic regression analysis of associations between baseline characteristics and acquiring housing adaptations over the follow-up (ELSA Waves 2–5 or 6–9), among men and women aged 65+ who reported no housing adaptations at relevant baseline wave (2 or 6).

	W2–5		W6–9	
	Men	Women	Men	Women
Variables measured at wave 2 or 6	OR [95% CI]	OR [95% CI]	OR [95% CI]	OR [95% CI]
Age	1.071^***^	1.033^**^	1.035^**^	1.030^*^
	[1.044,1.099]	[1.010,1.057]	[1.010,1.061]	[1.006,1.054]
Latent physical health tertile at baseline (Ref. Bad)				
Fair	0.880	0.698^*^	0.839	0.808
	[0.631,1.228]	[0.516,0.944]	[0.604,1.166]	[0.588,1.109]
Good	0.658^*^	0.543^***^	1.042	0.734
	[0.441,0.980]	[0.384,0.769]	[0.725,1.497]	[0.519,1.040]
Number of ADL limitations	1.254^*^	1.329^**^	1.458^*^	1.412^*^
	[1.018,1.544]	[1.099,1.606]	[1.088,1.953]	[1.066,1.871]
Number of IADL limitations	1.222	1.212	1.013	1.114
	[0.968,1.542]	[0.995,1.477]	[0.767,1.337]	[0.858,1.447]
Renter (Ref. Owner-occupier)	1.396	2.996^**^	0.789	0.721
	[0.529,3.684]	[1.428,6.285]	[0.361,1.726]	[0.335,1.554]
Unpartnered (Ref. Married/partnered)	0.588^**^	0.823	0.823	0.671^**^
	[0.412,0.838]	[0.626,1.081]	[0.604,1.122]	[0.511,0.881]
Educational attainment (Ref. Lower)				
Secondary	0.801	1.262	1.013	1.057
	[0.568,1.130]	[0.901,1.768]	[0.702,1.462]	[0.768,1.455]
Higher	0.647^*^	1.400	0.948	1.389
	[0.435,0.963]	[0.964,2.032]	[0.652,1.379]	[0.962,2.006]
Foreign/other	1.108	0.867	0.825	0.775
	[0.627,1.959]	[0.585,1.285]	[0.468,1.456]	[0.530,1.132]
Wealth quintile (Ref. Lowest quintile)				
4th quintile	1.048	1.927	1.255	0.606
	[0.389,2.823]	[0.921,4.031]	[0.560,2.812]	[0.280,1.313]
3rd quintile	0.854	1.390	1.098	0.667
	[0.305,2.392]	[0.637,3.033]	[0.485,2.487]	[0.307,1.450]
2nd quintile	1.080	1.441	1.391	0.835
	[0.389,2.996]	[0.658,3.160]	[0.617,3.137]	[0.375,1.860]
Highest quintile	0.853	1.041	1.298	0.661
	[0.302,2.406]	[0.464,2.336]	[0.568,2.965]	[0.295,1.480]
Constant	0.003^***^	0.028^***^	0.086^*^	0.283
	[0.000,0.028]	[0.004,0.117]	[0.012,0.648]	[0.040,1.973]
Observations	1182	1330	940	1086
Pseudo *R*^2^	0.071	0.071	0.021	0.033

Notes: Data source: English Longitudinal Study of Ageing Waves 2–9. ADL, Activities of Daily Living; IADL, Instrumental Activities of Daily Living; OR, odds ratio; CI, confidence intervals. ^*^  *P* < .05, ^**^  *P* < .01, ^***^  *P* < .001.

Table 4 shows results from mixed-effect models of associations between acquiring housing adaptations and health outcomes for men and women across ELSA Waves 2–5, with interaction terms (housing adaptations × latent health tertile at Wave 2). Acquiring housing adaptations was associated with an increase in the number of IADL limitations for men with initially bad latent health (coef. = 0.523 for the main effect, 95% CI: 0.343–0.702). However, for men with good latent health at baseline, acquiring housing adaptations was associated with a smaller increase (i.e. slower development) in the number of IADL limitations (coef. = −0.445 for the interaction term, 95% CI: −0.740 to −0.149). For women, acquiring housing adaptations was associated with a greater decrease in CASP for women with fair latent health (coef. = −1.334 for the interaction term, 95% CI: −2.581 to −0.088). We found no other interactive effects among men or women. Margins plots for these significant interactive effects are shown in Fig. S3.

**Table 4:** Results from mixed-effects models of the association between acquiring housing adaptations and multiple outcomes across ELSA Waves 2–5 for men (*N* = 1224) and women (*N* = 1363) who reported no housing adaptations at Wave 2, with interaction terms (housing adaptations × latent health tertile at Wave 2).

	CASP	Depressive symptoms	Number of ADL limitations	Number of IADL limitations	Fall
	coef. [95% CI]	coef. [95% CI]	coef. [95% CI]	coef. [95% CI]	coef. [95% CI]
**Men**					
Fixed-effects portion					
Housing adaptations (no = 0)	−0.848	0.088	0.269^***^	0.523^***^	0.429^*^
	[−1.949,0.252]	[−0.129,0.306]	[0.110,0.428]	[0.343,0.702]	[0.022,0.836]
Latent health tertile at Wave 2					
Bad (ref)					
Fair	2.006^***^	−0.188^*^	−0.156^***^	−0.184^***^	−0.435^**^
	[0.952,3.060]	[−0.371,−0.006]	[−0.239,−0.074]	[−0.262,−0.107]	[−0.740,−0.130]
Good	3.477^***^	−0.374^***^	−0.243^***^	−0.232^***^	−0.540^**^
	[2.353,4.600]	[−0.572,−0.177]	[−0.332,−0.155]	[−0.316,−0.148]	[−0.869,−0.211]
Housing adaptations × fair latent health	−0.966	0.0311	0.135	−0.176	0.0570
	[−2.507,0.576]	[−0.276,0.339]	[−0.094,0.365]	[−0.436,0.085]	[−0.531,0.645]
Housing adaptations × good latent health	−0.019	0.106	−0.134	**−0.445** ^^**^^	−0.057
	[−1.672,1.634]	[−0.232,0.445]	[−0.394,0.126]	**[−0.740,−0.149]**	[−0.722,0.609]
Random-effects portion					
Individual					
var(housing adaptations)	9.179	0.219	0.615	0.983	
	[5.172,16.291]	[0.077,0.625]	[0.491,0.770]	[0.818,1.182]	
var(constant)	41.167	1.130	0.196	0.188	1.704
	[37.246,45.500]	[1.008,1.266]	[0.171,0.225]	[0.164,0.215]	[1.316,2.208]
Var(Residual)	17.540	1.236	0.339	0.264	
	[16.472,18.677]	[1.170,1.306]	[0.321,0.358]	[0.249,0.279]	
Observations	3359	4026	4112	4112	4042
**Women**					
Fixed-effects portion					
Housing adaptations (no = 0)	−0.531	0.343^**^	0.286^***^	0.366^***^	0.520^**^
	[−1.421,0.360]	[0.114,0.572]	[0.156,0.417]	[0.216,0.516]	[0.198,0.842]
Latent health tertile at wave 2					
Bad (ref)					
Fair	2.250^***^	−0.323^**^	−0.160^***^	−0.169^***^	−0.023
	[1.215,3.286]	[−0.533,−0.113]	[−0.240,−0.079]	[−0.252,−0.085]	[−0.282,0.236]
Good	3.760^***^	−0.445^***^	−0.218^***^	−0.296^***^	−0.073
	[2.659,4.861]	[−0.672,−0.218]	[−0.305,−0.131]	[−0.387,−0.206]	[−0.353,0.206]
Housing adaptations × fair latent health at w2	−1.334^*^	−0.138	−0.000	0.069	−0.338
	[−2.581,−0.088]	[−0.470,0.195]	[−0.194,0.193]	[−0.155,0.293]	[−0.812,0.135]
Housing adaptations × good latent health at w2	−0.336	−0.194	−0.117	−0.218	−0.155
	[−1.615,0.943]	[−0.552,0.164]	[−0.327,0.094]	[−0.461,0.025]	[−0.670,0.359]
Random-effects portion					
Individual					
var(housing adaptations)	4.315	0.441	0.602	0.884	
	[1.769,10.530]	[0.211,0.924]	[0.501,0.724]	[0.745,1.048]	
var(constant)	43.188	1.579	0.229	0.248	1.280
	[39.347,47.405]	[1.416,1.760]	[0.202,0.260]	[0.219,0.282]	[1.001,1.639]
Var(Residual)	16.673	1.904	0.289	0.315	
	[15.683,17.725]	[1.807,2.006]	[0.274,0.305]	[0.299,0.332]	
Observations	3645	4555	4652	4652	4572

Notes: Data source: English Longitudinal Study of Ageing Waves 2–9. CASP, Control, Autonomy, Self-realisation and Pleasure; ADL, Activities of Daily Living; IADL, Instrumental Activities of Daily Living; CI, confidence intervals. All models adjusted for age, age squared, marital status, education, housing tenure and wealth quintile. ^*^  *P* < .05, ^**^  *P* < .01, ^***^  *P* < .001.

Table 5 presents results from the equivalent analysis for men and women across ELSA Waves 6–9, with interaction terms (housing adaptations × latent health tertile at Wave 6). Acquiring housing adaptations was associated with an increase in the number of ADL limitations for men with bad latent health (coef. = 0.537, 95% CI: 0.404–0.670), but with a smaller increase in ADL disability for men with fair latent health (coef. = −0.375 for the interaction term, 95% CI: −0.562 to −0.189) or good latent health (coef. = −0.448 for the interaction term, 95% CI: −0.632 to −0.265). Similar mitigating effects on ADL disability were found for women with good latent health (coef. = −0.151 for the interaction term, 95% CI: −0.278 to −0.024). Acquiring housing adaptations was also associated with a smaller increase in the number of IADL limitations for men with fair latent health (coef. = −0.238 for the interaction term, 95% CI: −0.431 to −0.044) or good latent health (coef. = −0.437 for the interaction term, 95% CI: −0.627 to −0.248) and for women with fair latent health (coef. = −0.174 for the interaction term, 95% CI: −0.323 to −0.025) or good latent health (coef. = −0.330 for the interaction term, 95% CI: −0.481 to −0.179). We did not find other interactive effects among men or women. Margins plots for these significant interactive effects are presented in Fig. S4.

**Table 5:** Results from mixed-effects models of the association between acquiring housing adaptations and multiple outcomes across ELSA Waves 6–9 for men (*N* = 983) and women (*N* = 1140) who reported no housing adaptations at Wave 6, with interaction terms (housing adaptations × latent health tertile at Wave 6).

	CASP	Depressive symptoms	Number of ADL limitations	Number of IADL limitations	Fall
	coef. [95% CI]	coef. [95% CI]	coef. [95% CI]	coef. [95% CI]	coef. [95% CI]
**Men**					
Fixed-effects portion					
Housing adaptations (no = 0)	−0.936^*^	0.216^*^	0.537^***^	0.509^***^	0.472^*^
	[−1.864,−0.008]	[0.034,0.393]	[0.404,0.670]	[0.372,0.647]	[0.109,0.835]
Latent health tertile at wave 6					
Bad (ref)					
Fair	2.040^***^	−0.265^**^	−0.063	−0.038	−0.336
	[0.851,3.229]	[−0.450,−0.080]	[−0.141,0.015]	[−0.114,0.0379]	[−0.689,0.017]
Good	3.575^***^	−0.443^***^	−0.129^**^	−0.103^*^	−0.607^**^
	[2.321,4.829]	[−0.640,−0.247]	[−0.213,−0.046]	[−0.185,−0.021]	[−0.984,−0.229]
Housing adaptations × fair latent health at w6	−0.063	0.100	**−0.375** ^^***^^	**−0.238** ^^*^^	−0.155
	[−1.329,1.204]	[−0.143,0.344]	**[−0.562,−0.189]**	**[−0.431,−0.044]**	[−0.658,0.347]
Housing adaptations × good latent health at w6	1.069	−0.144	**−0.448** ^^***^^	**−0.437** ^^***^^	−0.258
	[−0.147,2.286]	[−0.378,0.090]	**[−0.632,−0.265]**	**[−0.627,−0.248]**	[−0.757,0.241]
Random-effects portion					
Individual					
var(housing adaptations)	10.878	0.398	0.632	0.698	
	[7.243,16.335]	[0.262,0.604]	[0.540,0.741]	[0.598,0.815]	
var(constant)	40.742	0.840	0.109	0.097	1.453
	[36.414,45.585]	[0.735,0.959]	[0.089,0.133]	[0.077,0.122]	[1.077,1.960]
Var(Residual)	20.479	0.917	0.265	0.271	
	[19.124,21.929]	[0.860,0.978]	[0.248,0.282]	[0.254,0.289]	
Observations	3048	3258	3394	3394	3265
**Women**					
Fixed-effects portion					
Housing adaptations (no = 0)	−0.528	0.232^*^	0.191^***^	0.368^***^	0.379^*^
	[−1.428,0.372]	[0.033,0.432]	[0.098,0.283]	[0.260,0.477]	[0.060,0.699]
Latent health tertile at Wave 6					
Bad (ref)					
Fair	2.181^***^	−0.318^**^	−0.166^***^	−0.165^***^	−0.363^*^
	[0.982,3.381]	[−0.542,−0.094]	[−0.245,−0.088]	[−0.252,−0.079]	[−0.673,−0.053]
Good	4.320^***^	−0.551^***^	−0.246^***^	−0.290^***^	−0.493^**^
	[3.062,5.579]	[−0.787,−0.315]	[−0.330,−0.163]	[−0.383,−0.197]	[−0.817,−0.169]
Housing adaptations × fair latent health at w6	1.164	−0.0205	−0.057	**−0.174** ^^*^^	−0.082
	[−0.041,2.369]	[−0.286,0.245]	[−0.182,0.068]	**[−0.323,−0.025]**	[−0.513,0.348]
Housing adaptations × good latent health at w6	1.076	−0.243	**−0.151** ^^*^^	**−0.330** ^^***^^	−0.205
	[−0.110,2.262]	[−0.508,0.023]	**[−0.278,−0.024]**	**[−0.481,−0.179]**	[−0.640,0.230]
Random-effects portion					
Individual					
var(housing adaptations)	8.339	0.276	0.210	0.392	
	[4.873,14.271]	[0.129,0.593]	[0.165,0.268]	[0.323,0.474]	
var(constant)	46.023	1.335	0.139	0.187	1.171
	[41.441,51.112]	[1.181,1.510]	[0.117,0.165]	[0.159,0.220]	[0.884,1.551]
Var(Residual)	25.788	1.647	0.272	0.296	
	[24.168,27.516]	[1.553,1.747]	[0.257,0.289]	[0.279,0.315]	
Observations	3507	3789	3907	3907	3800

Data source: English Longitudinal Study of Ageing Waves 2–9. CASP, Control, Autonomy, Self-realisation and Pleasure; ADL, Activities of Daily Living; IADL, Instrumental Activities of Daily Living; CI, confidence intervals. All models adjusted for age, age squared, marital status, education, housing tenure and wealth quintile. ^*^  *P* < .05, ^**^  *P* < .01, ^***^  *P* < .001.

### Sensitivity analysis

We re-estimated the mixed-effect models using alternative moderators: latent health quartile (Table S3), the continuous form of latent health (Table S4) and self-reported mobility impairments [22] [Figs S5 and S6, although this had poor concordance with latent physical health (Fig. S2)], and the findings in Tables 4 and 5 held. To compare our findings on falls with prior work [22], we drew margins plots using three moderators: latent health tertile (Fig. S7), number of mobility impairments (Fig. S8) and categorical mobility impairments (Fig. S9). The mitigating effect of housing adaptations on falls was not only greater for those with worse mobility impairments, largely consistent with Chandola and Rouxel [22], but also greater for those with better latent health. This discrepancy in findings may be driven by dissimilar sample selection (gender-stratified and cohort-specific in our study), different operationalisation of housing adaptations (no distinction between internal and external adaptations in our analysis; no consideration of changes in question wording at Wave 6 of ELSA in the earlier study [22]), alternate models (mixed effect rather than fixed effect) and discordance between latent health status based on observer-measured indicators and self-reported mobility impairments. Regardless of which moderator is examined, the findings on falls need to be interpreted cautiously because ELSA respondents reported falls that happened in the last 2 years, so these might predate the installation of housing adaptations.

Due to the longitudinal design of ELSA, those reporting no housing adaptation at Wave 6 may have already been interviewed in the earlier cohort. We therefore excluded those interviewed twice (313 men and 371 women) and re-estimated mixed-effect models (Table S5). Our findings in Tables 4 and 5 held except for interactive effects on women’s ADLs and IADLs in later cohorts.

## Discussion

Using nationally representative data from ELSA Waves 2–9, we examined factors associated with acquiring housing adaptations among older men and women from earlier (Waves 2–5) and later (Waves 6–9) cohorts who reported no housing adaptations at baseline waves. Secondly, we examined how participants’ initial latent physical health (derived from objective measures obtained during nurse visits) moderated associations between acquiring housing adaptations and health outcomes.

As suggested by prior work [22], we tried factor analysis but did not find a strong statistical justification to distinguish between internal (e.g. bathroom adaptations) and external (e.g. widened doorways) modifications. Descriptive results showed that the incidence of acquiring housing adaptations was slightly higher among women than men, possibly due to women’s greater needs for performance-enhancing environments given female disadvantages in health [25]. The incidence was markedly higher in the later cohort; however, this may reflect changes in question wording, as well as changes in standards and expectations. More ADL limitations and older age were generally positively associated with higher odds of acquiring housing adaptations. A recent English Housing Survey report [8] found that it was those with mid-level incomes who were the least likely to have adaptations, possibly because they may be less likely to have access to help from local authorities or to means-tested DFGs than those on low incomes and be less able to afford modifications themselves than those on higher incomes. However, we found no consistent socio-economic gradient in acquiring adaptations by education, tenure or wealth.

We found that housing adaptations were associated with slower development of IADL/ADL disability among men and women with initially good latent physical health, differing from some previous studies [23, 24] which reported that beneficial effects were greater for people with poor or moderate health. Our findings suggest that housing adaptations may have preventive effects and slow down the development of disability in those with good baseline latent physical health, consistent with Peace and Darton [36] and several other small-scale studies emphasising home modifications should be made as soon as a need has been identified [37]. With these adaptations in place, people with initially good latent physical health may be able to preserve their functional capabilities and benefit from a slower health decline. However, we also found that acquiring housing adaptations was associated with a greater decrease in CASP for women in the earlier cohort with fair latent health compared with women with bad latent health. Qualitative studies have found that older people dislike anything in the home that looks medical and indicative of a disability, and this may perhaps particularly apply to those who perceive less need for adaptation [38]. For this and other reasons, it is very important to involve older people and their families in decisions about adaptations [10, 38]. Sensitivity analyses using self-reported mobility impairments as an alternative moderator led to different results from our main finding but similar results to those of Chandola and Rouxel [22]. We discussed possible reasons for this discrepancy in the sensitivity analysis.

The contributions of this study are threefold. Firstly, we used a latent physical health variable derived from observer-measured indicators to reduce self-report bias and illustrated the discordance between results using this measure and a self-reported measure of mobility impairments. Secondly, we examined the factors associated with acquiring housing adaptations before investigating the effects of housing adaptations on participants’ health outcomes. Thirdly, by dividing participants into earlier and later cohorts, we showed that the change in the wording/design of the ELSA questionnaire might lead to overestimation of increases in the incidence of housing adaptations.

The study has some limitations. ELSA is recognised as a high-quality longitudinal study of the older population of England, but nonresponse and attrition may affect representativeness [39], as suggested by slight differences in tenure distribution compared with national census data, and our modelling strategy meant that we were unable to use the survey weights. We lacked information on whether adaptations were paid for from DFGs, by local authorities or by respondents themselves and could not assess whether any tenure group used DFGs disproportionately. Additionally, we cannot establish the causal moderating effect of latent health on the associations between housing adaptations and health outcomes in this observational study. Also, we were unable to consider cost cost-effectiveness of adaptations. In the UK context, there is evidence that the costs of overall housing improvements (not specifically adaptations) are offset by savings in health care costs [40, 41] and also evidence of the cost-effectiveness of some housing-related falls prevention interventions [40]. However, evidence on the cost-effectiveness of the kind of adaptations considered here is inconclusive [42] and this remains a research gap [43]. Nevertheless, these results, and particularly findings suggesting preventive effects of housing adaptations on the development of disability, support calls made by others for greater recognition, awareness and implementation of housing adaptations as a means of promoting the functional ability of older people [10, 44].

## Supplementary Material

aa-24-0757-File002_afaf023

## Data Availability

This study analyses data from the English Longitudinal Study of Ageing (ELSA), which is publicly available upon registration. Code-related inquiries should be directed to the corresponding author.
