# Supplementary material for: Housing adaptations and older adults’ health trajectories by level of initial health: evidence from the English Longitudinal Study of Ageing
Source: Age Ageing. 2025 Feb 17;54(2):afaf023. doi: 10.1093/ageing/afaf023 (PMC11831028; doi:10.1093/ageing/afaf023)
Supplement: aa-24-0757-File002_afaf023 [file aa-24-0757-file002_afaf023.docx]

**Supplementary Materials for manuscript '**Housing adaptations and older adults’ health trajectories by level of initial health: Evidence from the English Longitudinal Study of Ageing**'**

Table of Contents

[Table S1. Standardised factor loadings for observer-measured variables, goodness of fit indicators and distribution of latent physical health variables for men and women at ELSA wave 2 and wave 6, corresponding to Tables 4 and 5. 2](#_Toc187964550)

[Figure S1. Factor scores for men's and women's latent physical health at ELSA waves 2 and 6. 2](#_Toc187964551)

[Table S2. Proportion with housing adaptations among those with no housing adaptations at first observation point in the relevant cohort (ELSA wave 2 or 6), by gender and response pattern. 3](#_Toc187964552)

[Figure S2. Concordance between latent health decile and self-reported mobility impairments for men and women at ELSA wave 2/6. The size of circles corresponds to frequencies. 4](#_Toc187964553)

[Figure S3. Margins plots for significant associations in Table 4. 5](#_Toc187964554)

[Figure S4. Margins plots for significant associations in Table 5. 5](#_Toc187964555)

[Figure S5. Margins plots for significant associations in Table 4 re-examined by using the number of mobility impairments as the alternative moderator. 6](#_Toc187964556)

[Figure S6. Margins plots for significant associations in Table 5 re-examined by using the number of mobility impairments as the alternative moderator. 6](#_Toc187964557)

[Figure S7. Margins plots for the effects of housing adaptations on falls moderated by initial latent health tertile. 7](#_Toc187964558)

[Figure S8. Margins plots for the effects of housing adaptations on falls moderated by initial number of mobility impairments 7](#_Toc187964559)

[Figure S9. Margins plots for the effects of housing adaptations on falls moderated by initial categories of mobility impairments. 8](#_Toc187964560)

[Table S3. Results from mixed-effects models re-examining the association in Tables 4 and 5, with alternative interaction terms (Housing adaptations × latent health quartile at waves 2/6) 9](#_Toc187964561)

[Table S4. Results from mixed-effects models re-examining the association in Tables 4 and 5, with alternative interaction terms (Housing adaptations × continuous latent health at waves 2/6) 11](#_Toc187964562)

[Table S5. Results from mixed-effects models re-examining the association in Tables 4 and 5, excluding those interviewed in both earlier and later cohorts 12](#_Toc187964563)

## Table S1. Standardised factor loadings for observer-measured variables, goodness of fit indicators and distribution of latent physical health variables for men and women at ELSA wave 2 and wave 6, corresponding to Tables 4 and 5.

|  | Wave 2 |  | Wave 6 |  |
| --- | --- | --- | --- | --- |
| Observed-measured variables | Men | Women | Men | Women |
| Maximum grip strength | 0.67 | 0.60 | 0.64 | 0.56 |
| Full tandem stand | 0.33 | 0.35 | 0.37 | 0.34 |
| Inversed chair rise time | 0.39 | 0.39 | 0.32 | 0.46 |
| Lung function (top FVC reading) | 0.50 | 0.56 | 0.58 | 0.48 |
| Goodness of fit indicators |  |  |  |  |
| RMSEA | 0.00 | 0.02 | 0.01 | 0.07 |
| CFI | 1.00 | 1.00 | 1.00 | 0.96 |
| TLI | 1.01 | 0.99 | 1.00 | 0.87 |
| Latent physical health |  |  |  |  |
| Range | (-13.42, 13.49) | (-7.17, 10.84) | (-13.88, 11.94) | (-7.00, 6.23) |
| Skewness | -0.10 | 0.18 | -0.26 | -0.20 |
| Kurtosis | 3.10 | 3.65 | 3.39 | 3.27 |
| Observations | 1,224 | 1,228 | 867 | 994 |

Notes: Data source: English Longitudinal Study of Ageing wave 2 and wave 6. RMSEA = root mean square error of approximation, CFI = comparative fit index, TLI = Tucker–Lewis index. Factor loadings were generally acceptable (all above 0.30). Goodness of fit indicators were satisfactory, except for RMSEA and TLI for women at wave 6.

## Figure S1. Factor scores for men's and women's latent physical health at ELSA waves 2 and 6.

## Table S2. Proportion with housing adaptations among those with no housing adaptations at first observation point in the relevant cohort (ELSA wave 2 or 6), by gender and response pattern.

|  | Men |  |  |  |  | Women |  |  |  |  |
| --- | --- | --- | --- | --- | --- | --- | --- | --- | --- | --- |
|  | w2 | w3 | w4 | w5 | N | w2 | w3 | w4 | w5 | N |
| **Earlier cohort** |  |  |  |  |  |  |  |  |  |  |
| Present at waves 2 3 | 0 | 0.24 |  |  | 156 | 0 | 0.23 |  |  | 152 |
| Present at waves 2 4 | 0 |  | 0.17 |  | 6 | 0 |  | 0.31 |  | 13 |
| Present at waves 2 5 | 0 |  |  | 0.23 | 23 | 0 |  |  | 0.38 | 25 |
| Present at waves 2 3 4 | 0 | 0.19 | 0.31 |  | 155 | 0 | 0.21 | 0.36 |  | 147 |
| Present at waves 2 3 5 | 0 | 0.16 |  | 0.26 | 44 | 0 | 0.22 |  | 0.31 | 42 |
| Present at waves 2 4 5 | 0 |  | 0.26 | 0.21 | 28 | 0 |  | 0.31 | 0.29 | 32 |
| Present at waves 2 3 4 5 | 0 | 0.12 | 0.17 | 0.23 | 812 | 0 | 0.17 | 0.22 | 0.30 | 952 |
| Total |  |  |  |  | 1,224 |  |  |  |  | 1,363 |
| **Later cohort** |  |  |  |  |  |  |  |  |  |  |
| Present at waves 6 7 | 0 | 0.32 |  |  | 97 | 0 | 0.45 |  |  | 130 |
| Present at waves 6 8 | 0 |  | 0.62 | 0.57 | 9 | 0 |  | 0.75 |  | 9 |
| Present at waves 6 9 | 0 |  |  | 0.67 | 4 | 0 |  |  | 1 | 3 |
| Present at waves 6 7 8 | 0 | 0.45 | 0.58 |  | 134 | 0 | 0.37 | 0.47 |  | 123 |
| Present at waves 6 7 9 | 0 | 0.26 |  | 0.47 | 19 | 0 | 0.26 |  | 0.45 | 23 |
| Present at waves 6 8 9 | 0 |  | 0.47 | 0.60 | 15 | 0 |  | 0.57 | 0.65 | 21 |
| Present at waves 6 7 8 9 | 0 | 0.36 | 0.47 | 0.57 | 705 | 0 | 0.37 | 0.50 | 0.61 | 831 |
| Total |  |  |  |  | 983 |  |  |  |  | 1,140 |

Notes: Data source: English Longitudinal Study of Ageing waves 2-9

## Figure S2. Concordance between latent health decile and self-reported mobility impairments for men and women at ELSA wave 2/6. The size of circles corresponds to frequencies.

## Figure S3. Margins plots for significant associations in Table 4.

## Figure S4. Margins plots for significant associations in Table 5.

## Figure S5. Margins plots for significant associations in Table 4 re-examined by using the number of mobility impairments as the alternative moderator.

## Figure S6. Margins plots for significant associations in Table 5 re-examined by using the number of mobility impairments as the alternative moderator.

## Figure S7. Margins plots for the effects of housing adaptations on falls moderated by initial latent health tertile.

Figure S8. Margins plots for the effects of housing adaptations on falls moderated by initial number of mobility impairments.

## Figure S9. Margins plots for the effects of housing adaptations on falls moderated by initial categories of mobility impairments.

## Table S3. Results from mixed-effects models re-examining the association in Tables 4 and 5, with alternative interaction terms (Housing adaptations × latent health quartile at waves 2/6)

|  | wave 2, men, IADLs | wave 2, women, CASP | wave 6, men, ADLs | wave 6, men, IADLs | wave 6, women, ADLs | wave 6, women, IADLs |
| --- | --- | --- | --- | --- | --- | --- |
|  | coef. [95%CI] | coef. [95%CI] | coef. [95%CI] | coef. [95%CI] | coef. [95%CI] | coef. [95%CI] |
| Housing adaptations (no = 0) | 0.512^***^ | -0.230 | 0.539^***^ | 0.464^***^ | 0.165^**^ | 0.412^***^ |
|  | [0.309,0.715] | [-1.265,0.804] | [0.383,0.695] | [0.303,0.626] | [0.058,0.271] | [0.287,0.537] |
| Latent health quartile at w2 or w6 (4th quartile (poorest) = ref) |  |  |  |  |  |  |
| 3rd quartile | -0.225^***^ | 2.550^***^ | -0.077 | -0.072 | -0.163^***^ | -0.169^***^ |
|  | [-0.313,-0.138] | [1.402,3.698] | [-0.164,0.010] | [-0.157,0.013] | [-0.251,-0.076] | [-0.265,-0.072] |
| 2nd quartile | -0.285^***^ | 3.670^***^ | -0.101^*^ | -0.149^**^ | -0.229^***^ | -0.280^***^ |
|  | [-0.378,-0.192] | [2.401,4.940] | [-0.196,-0.007] | [-0.241,-0.056] | [-0.327,-0.131] | [-0.388,-0.172] |
| 1st quartile (healthiest) | -0.319^***^ | 4.938^***^ | -0.140^**^ | -0.138^**^ | -0.289^***^ | -0.348^***^ |
|  | [-0.417,-0.221] | [3.658,6.218] | [-0.237,-0.043] | [-0.233,-0.043] | [-0.388,-0.190] | [-0.458,-0.239] |
| Housing adaptations × latent health quartile at w2 or w6 |  |  |  |  |  |  |
| Housing adaptations × 3rd quartile latent health at w2 or w6 | -0.0181 | **-1.611^*^** | **-0.233^*^** | -0.084 | 0.015 | -0.178^*^ |
|  | [-0.302,0.266] | **[-2.996,-0.226]** | **[-0.442,-0.024]** | [-0.301,0.133] | [-0.126,0.155] | [-0.345,-0.011] |
| Housing adaptations × 2nd quartile latent health at w2 or w6 | **-0.409^*^** | -0.546 | **-0.405^***^** | -0.231 | -0.055 | **-0.287^**^** |
|  | **[-0.743,-0.075]** | [-2.063,0.971] | **[-0.628,-0.183]** | [-0.461,0.000] | [-0.205,0.095] | **[-0.466,-0.109]** |
| Housing adaptations × 1st quartile latent health at w2 or w6 | **-0.433^*^** | -1.079 | **-0.474^***^** | **-0.421^***^** | -0.137 | **-0.385^***^** |
|  | **[-0.768,-0.098]** | [-2.562,0.405] | **[-0.690,-0.259]** | **[-0.645,-0.198]** | [-0.286,0.011] | **[-0.560,-0.209]** |
| Observations | 4,112 | 3,645 | 3,394 | 3,394 | 3,907 | 3,907 |

Notes: Data source: CASP = Control, Autonomy, Self-realisation and Pleasure; ADL = Activities of Daily Living; IADL = Instrumental Activities of Daily Living; CI = confidence intervals. All models adjusted for age, age squared, marital status, education, housing tenure, and wealth quintile. Only results from fixed-effect portion were reported. * p < 0.05, ** p < 0.01, *** p < 0.001.

## Table S4. Results from mixed-effects models re-examining the association in Tables 4 and 5, with alternative interaction terms (Housing adaptations × continuous latent health at waves 2/6)

|  | wave 2, men, IADLs | wave 2, women, CASP | wave 6, men, ADLs | wave 6, men, IADLs | wave 6, women, ADLs | wave 6, women, IADLs |
| --- | --- | --- | --- | --- | --- | --- |
|  | coef. [95%CI] | coef. [95%CI] | coef. [95%CI] | coef. [95%CI] | coef. [95%CI] | coef. [95%CI] |
| Housing adaptations (no = 0) | 0.319^***^ | -1.078^***^ | 0.264^***^ | 0.282^***^ | 0.273^***^ | 0.200^***^ |
|  | [0.200,0.438] | [-1.631,-0.525] | [0.184,0.343] | [0.201,0.364] | [0.134,0.412] | [0.134,0.267] |
| Latent health at w2 or w6 | -0.031^***^ | 0.762^***^ | -0.016^***^ | -0.018^***^ | -0.122^***^ | -0.066^***^ |
|  | [-0.040,-0.022] | [0.568,0.956] | [-0.026,-0.007] | [-0.026,-0.009] | [-0.164,-0.081] | [-0.085,-0.046] |
| Housing adaptations × latent health at w2 or w6 | **-0.038^*^** | -0.080 | **-0.055^***^** | **-0.050^***^** | **-0.076^*^** | **-0.073^***^** |
|  | **[-0.067,-0.008]** | [-0.315,0.155] | **[-0.075,-0.035]** | **[-0.070,-0.029]** | **[-0.139,-0.012]** | **[-0.103,-0.043]** |
| Observations | 4,112 | 3,645 | 3,394 | 3,394 | 3,907 | 3,907 |

Notes: Data source: CASP = Control, Autonomy, Self-realisation and Pleasure; ADL = Activities of Daily Living; IADL = Instrumental Activities of Daily Living; CI = confidence intervals. All models adjusted for age, age squared, marital status, education, housing tenure, and wealth quintile. Only results from fixed-effect portion were reported. * p < 0.05, ** p < 0.01, *** p < 0.001.

Table S5. Results from mixed-effects models re-examining the association in Tables 4 and 5, excluding those interviewed in both earlier and later cohorts.

|  | Earlier cohort only, men, IADLs | Earlier cohort only, women, CASP | Later cohort only, men, ADLs | Later cohort only, men, IADLs | Later cohort only, women, ADLs | Later cohort only, women, IADLs |
| --- | --- | --- | --- | --- | --- | --- |
|  | coef. [95%CI] | coef. [95%CI] | coef. [95%CI] | coef. [95%CI] | coef. [95%CI] | coef. [95%CI] |
| Housing adaptations (no = 0) | 0.573^***^ | -0.197 | 0.462^***^ | 0.380^***^ | 0.097 | 0.163^**^ |
|  | [0.367,0.780] | [-1.180,0.787] | [0.307,0.618] | [0.231,0.529] | [-0.003,0.197] | [0.053,0.272] |
| Latent health at w2 or w6 (poor = ref) |  |  |  |  |  |  |
| Fair | -0.229^***^ | 2.751^***^ | -0.165^***^ | -0.054 | -0.188^***^ | -0.198^***^ |
|  | [-0.330,-0.128] | [1.507,3.995] | [-0.246,-0.083] | [-0.136,0.029] | [-0.264,-0.111] | [-0.284,-0.111] |
| Good | -0.325^***^ | 4.440^***^ | -0.157^***^ | -0.088^*^ | -0.203^***^ | -0.260^***^ |
|  | [-0.435,-0.215] | [3.093,5.787] | [-0.242,-0.071] | [-0.176,-0.001] | [-0.283,-0.122] | [-0.351,-0.170] |
| Housing adaptations × latent health at w2 or w6 |  |  |  |  |  |  |
| Housing adaptations × fair health | -0.206 | **-1.612^*^** | **-0.334^**^** | -0.183 | 0.105 | 0.034 |
|  | [-0.507,0.095] | **[-2.969,-0.255]** | **[-0.549,-0.118]** | [-0.389,0.023] | [-0.033,0.244] | [-0.119,0.186] |
| Housing adaptations × good health | **-0.475^**^** | -0.719 | **-0.424^***^** | **-0.360^***^** | -0.057 | -0.139 |
|  | **[-0.801,-0.149]** | [-2.072,0.633] | **[-0.639,-0.210]** | **[-0.565,-0.155]** | [-0.196,0.083] | [-0.293,0.015] |
| N | 911 | 992 | 670 | 670 | 769 | 769 |
| Observations | 2,937 | 2,484 | 2,353 | 2,353 | 2,685 | 2,685 |

Notes: Data source: CASP = Control, Autonomy, Self-realisation and Pleasure; ADL = Activities of Daily Living; IADL = Instrumental Activities of Daily Living; CI = confidence intervals. All models adjusted for age, age squared, marital status, education, housing tenure, and wealth quintile. Only results from fixed-effect portion were reported. * p < 0.05, ** p < 0.01, *** p < 0.001
